# Bridging the Gap between Literature and Practice: Nationwide Outcomes of Endoscopic Ultrasound-guided Hepatico-gastrostomy

**DOI:** 10.1055/a-2884-9422

**Published:** 2026-06-25

**Authors:** David M. de Jong, Lydi M.J.W. van Driel, Jurriën G.P. Reijnders, Akin Inderson, Jan Werner Poley, Roy L.J. van Wanrooij, Rogier P. Voermans, Paul Didden, Thomas R. de Wijkerslooth, Foke van Delft, Niels G. Venneman, Robert C. Verdonk, Johanna P. van Nes, Marco J. Bruno, Willem J. Lammers

**Affiliations:** 1 Department of Gastroenterology and HepatologyErasmus MC University Medical CenterRotterdamThe Netherlands; 2 Department of Gastroenterology and Hepatology 4501 Leiden University Medical CenterLeidenThe Netherlands; 3 Department of Gastroenterology and HepatologyMaastricht University Medical CenterMaastrichtThe Netherlands; 4 Gastroenterology and HepatologyAmsterdam UMCAmsterdamNoord-HollandThe Netherlands; 5 Cancer Centre AmsterdamAmsterdamThe Netherlands; 6 Gastroenterology and Hepatology 8124 University Medical Centre UtrechtUtrechtThe Netherlands; 7 Gastroenterology and HepatologyAntoni van Leeuwenhoek ZiekenhuisAmsterdamThe Netherlands; 8 Gastroenterology and Hepatology 6034 Radboud University Medical CenterNijmegenThe Netherlands; 9 Gastroenterology and HepatologyMedical Spectrum TwenteEnschedeThe Netherlands; 10 Gastroenterology and Hepatology 6028 Sint Antonius HospitalNieuwegeinThe Netherlands

**Keywords:** endoscopic ultrasound, hepatico-gastrostomy, malignant biliary obstruction

## Abstract

**Background** Endoscopic ultrasound-guided hepatico-gastrostomy (EUS-HGS) enables internal biliary drainage via the stomach in patients with altered anatomy, inadequate left hepatic duct drainage, or duodenal obstruction. The technically demanding procedure is only performed at expert centers in the Netherlands. Most available evidence originates from selected cohorts, overestimating real-world outcomes. We evaluated outcomes of EUS-HGS in a nationwide cohort.

**Methods** This nationwide retrospective cohort study included all patients in the Netherlands who underwent EUS-HGS attempts between 2009 and 2025, outside of prospective studies. The primary outcome was technical success. Secondary outcomes included clinical success, complications, time to recurrent biliary obstruction (RBO), and overall survival.

**Results** A total of 107 procedures in 105 patients were analyzed (median age: 68 years [IQR: 59–76]; 55%: male). Technical success was achieved in 77% (81/105). Failures were due to absence of a safe puncture tract ( *n* = 7), unsuccessful bile duct puncture ( *n* = 3), inability to achieve deep guidewire insertion ( *n* = 3), failed fistula formation ( *n* = 3), or unsuccessful or nonattempted stent placement ( *n* = 7). Clinical success occurred in 78% of technically successful cases (63/81), corresponding to 60% in intention-to-treat analysis (63/105). Procedural complications occurred in 16/107 (15%), and postprocedural complications in 17/107 (16%). One procedure-related death occurred. RBO occurred in 28% (23/81) during a median follow-up of 61 days [IQR: 23–131]. Median overall survival was 91 days [IQR: 66–155].

**Conclusion** In this nationwide real-world cohort, technical and clinical success rates of EUS-HGS were modest compared with selected expert series, underscoring the need for prospective studies to improve patient selection, stent strategies, and procedural safety.

## Introduction

In patients with malignant biliary obstruction (MBO), endoscopic retrograde cholangio-pancreatography (ERCP) with stent placement is the first-line approach for biliary drainage. 1 When ERCP is unsuccessful, three alternatives exist: surgical biliary reconstruction, percutaneous trans-hepatic biliary drainage (PTBD), or endoscopic ultrasound (EUS)–guided drainage. The most appropriate method depends on therapeutic intent, obstruction level, availability of local expertise and equipment, and the patient’s overall condition. 2

Several studies have demonstrated clear advantages of EUS-guided techniques over PTBD, 3 and they are now recommended in the 2022 European Society of Gastrointestinal Endoscopy (ESGE) guideline on therapeutic EUS. 4 EUS-guided techniques offers higher clinical success, lower complication rates, longer stent patency, and improved quality of life. 3 5 6

The main EUS-guided techniques are hepatico-gastrostomy (EUS-HGS) and choledocho-duodenostomy (EUS-CDS), each with different indications. EUS-CDS is best suited for distal obstructions, whereas EUS-HGS is preferred in patients with left-sided obstruction, (malignant) gastric-outlet obstruction treated with gastro-enterostomy, or surgically altered anatomy, and is increasingly used as a complementary approach for unresectable hilar obstruction. 2 7 Other EUS-based options include gallbladder drainage and EUS-guided rendezvous procedures in selected cases. Evidence supporting EUS-HGS has expanded alongside the development of dedicated self-expandable metal stents (SEMSs). 8 9 However, much of the literature is limited by study design and restrictive inclusion criteria. Many studies include only patients in whom bile duct puncture or stent placement was attempted or achieved, excluding cases where puncture was not pursued. Together with small sample sizes and highly selected patient populations from expert centers, this may overestimate real-world outcomes.

The primary objective of this study is to evaluate the technical success rates of EUS-HGS across all Dutch hospitals. Secondary objectives include describing clinical success, complications, time to recurrent biliary obstruction (RBO), overall survival, and nationwide practice patterns, with particular emphasis on stent selection, within a comprehensive national cohort.

## Patients and Methods

### Study Population

A nationwide, multicenter, retrospective study was performed in all nine centers that performed at least one EUS-HGS attempt between December 2009 and August 2025 ( **Supplementary Table 1** ). All centers obtained ethical approval from their local ethics committees (MEC-2024-0700). The study was conducted according to the STROBE guidelines. 10

Patients were identified from local endoscopy databases (Endobase or Clinical Assistant) or prospectively maintained lists of EUS-guided interventions. All patients who underwent attempted EUS-HGS were eligible, excluding those younger than 18 years. Patients included in prospective studies were excluded.

### Hepatico-gastrostomy Procedure

**Video 1** EUS-HGS performed with pcSEMS in a patient with malignant biliary obstruction.


No standardized national protocol for EUS-HGS was available during the study period. However, in general, the procedure followed a common sequence reflecting standard practice in participating centers. 11 Cross-sectional imaging with computed tomography (CT) and/or magnetic resonance imaging (MRI) was performed before EUS-HGS attempt. Cases were discussed in multidisciplinary team meetings to determine the optimal biliary drainage approach.

Early procedures (circa 2010) were often performed under conscious sedation, whereas more recent procedures were conducted under propofol sedation. Anticoagulant therapy was discontinued when feasible.

Although no formal protocol was used, the procedure generally followed these steps.

1. The intrahepatic bile ducts of the left liver lobe were identified and ductal dilatation was assessed to determine suitability for puncture, at the discretion of the endoscopist.
2. When adequate dilatation was present, an intrahepatic bile duct (segment 2 or 3) was punctured using a 19 Gauge fine-needle aspiration (FNA) needle. Aspiration of bile confirmed adequate intraductal positioning, followed by contrast injection to obtain a cholangiogram.
3. A guidewire was advanced through the needle, preferably into the extrahepatic bile duct.
4. A 6-French cystotome was advanced over the guidewire to create the fistula. In selected cases, a 4 mm dilatation balloon was used instead.
5. Stent selection was up to the discretion of the endoscopists. Early procedures typically involved placement of an uncovered SEMS within the bile duct, followed by deployment of a fully covered SEMS (WallFlex; Boston Scientific, Natick, MA, USA) across the fistula to reduce bile leakage risk. In later years, a dedicated partially covered SEMS (Giobor; Taewoong, Seoul, Republic of Korea) was preferentially used.

To reduce the risk of stent migration, an endoscopic clip could be placed at the discretion of the endoscopist. When the intragastric portion was <3 cm, the stent was gently repositioned with grasping forceps or an additional stent was deployed. Antibiotic prophylaxis was administered according to local protocols. An example of an EUS-HGS procedure is shown in Video 1 .

### Data Collection

Data were collected retrospectively from electronic patient records on demographic factors (sex, age), medical history, World Health Organization (WHO) performance status, etiology of the MBO, underlying disease (staging), preprocedure cross-sectional imaging, and previous diagnostic work-up, treatment, and biliary drainage attempts. Laboratory variables before the procedure were also collected. EUS-HGS data comprised technical success of EUS-HGS and procedural complications. Post-EUS-HGS data included complications, RBO, RBO-related interventions, and survival.

### Outcome Definition

The primary outcome was technical success of EUS-HGS, defined as suitable stent positioning confirmed by endoscopy and/or radiography. Clinical success of EUS-HGS was defined as no need for additional biliary drainage interventions during follow-up. Other secondary outcomes included procedural and postprocedural complications, graded according to the AGREE classification, 12 and overall survival.

### Statistical Analysis

Patient characteristics were summarized using descriptive characteristics. Categorical variables were presented as frequencies with percentages, and continuous variables as means with standard deviations if normally distributed or medians with interquartile ranges (IQRs) otherwise.

Overall survival was analyzed with the Kaplan–Meier method. Survival curves were compared with the log-rank test. Cox proportional hazards regression was used for univariable and multivariable survival analyses. RBO was modeled as a time-varying covariate using the counting-process approach. Results were reported as hazard ratios (HRs) with 95% confidence intervals (CIs).

For competing risk analysis, cumulative incidence of RBO was estimated with death as a competing event. Cumulative incidence functions were plotted for RBO and death, and Gray’s test compared groups. Fine and Gray proportional subdistribution hazards regression estimated subdistribution hazard ratios (SHRs) with 95% CIs for predictors of RBO. Based on prior literature and clinical rationale, stricture location (proximal vs distal) was identified as a confounder in the adjusted model. A two-sided *P* -value of <0.05 considered statistically significant. Analyses were conducted using R (version 4.3.1.).

## Results

### Study Population and Baseline Characteristics

**Fig. 1:**
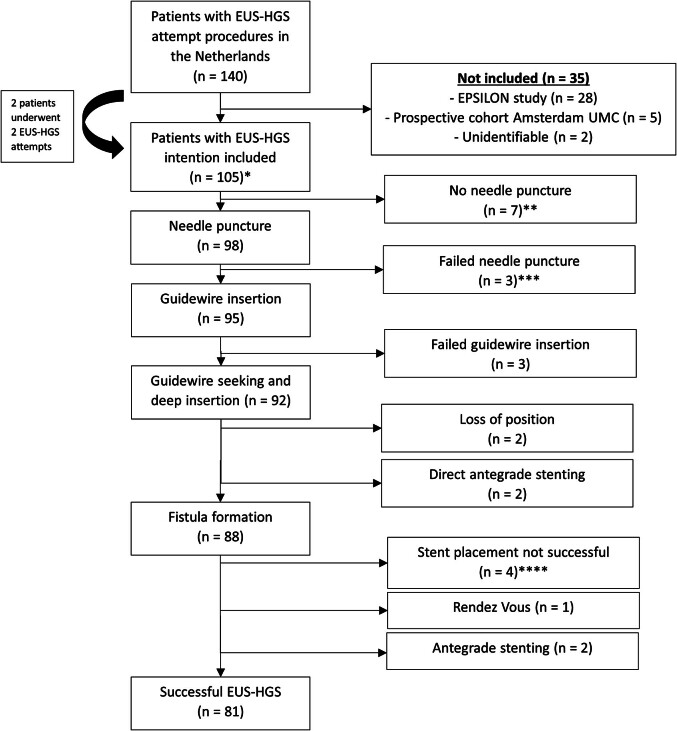
Flowchart of the included patients in which intention was to perform EUS-HGS in the Netherlands * = One patient underwent two EUS procedures without puncture attempts due to insufficient bile duct dilatation, whereas another patient underwent successful EUS-HGS after an initial EUS showing inadequate ductal dilatation. The second procedure for both patients is taken into account in the flowchart. ** = Inadequate bile duct dilatation ( *n* = 4), perihepatic ascites ( *n* = 2), and unstable scope position ( *n* = 1). *** = no clear cholangiogram after contrast injection ( *n* = 2), and portal vein puncturing ( *n* = 1) **** = abandoned due to cardiac arrest ( *n* = 1), dislocation at moment of stent insertion ( *n* = 2) or before stent insertion ( *n* = 1).

**Fig. 2:**
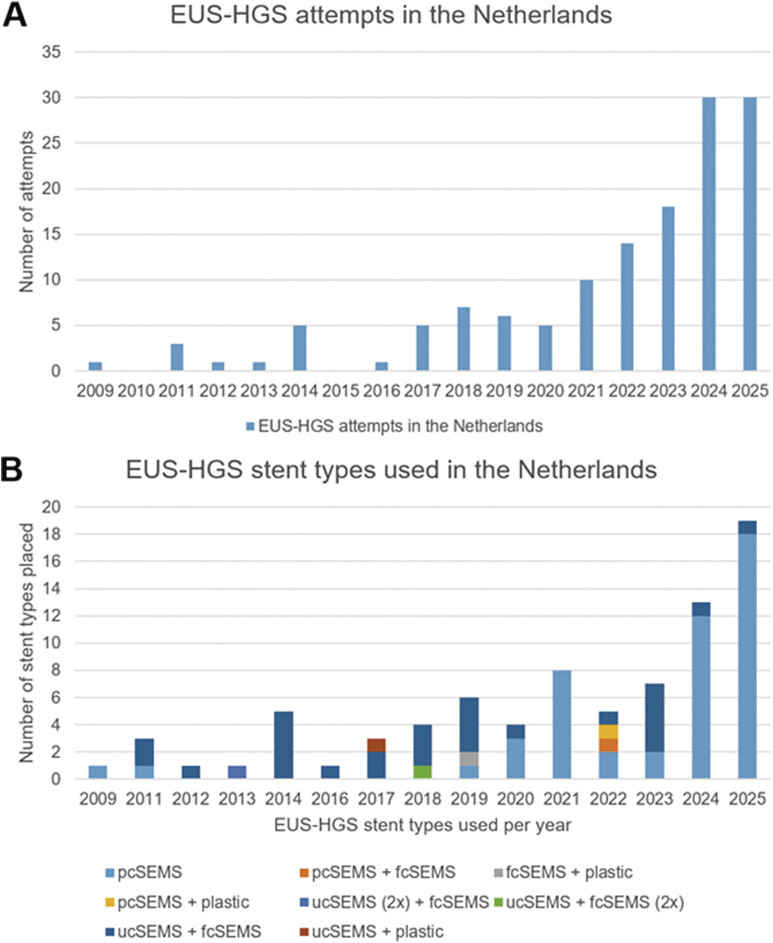
( **A** ) Overall trend of EUS-HGS attempts in the Netherlands over time (up to 30-08-2025). Patients excluded from this dataset include not identifiable ( *n* =–2), EPSILON ( *n* = 28), and prospective study ( *n* = 5). ( **B** ) Overall trend of stent types used in the Netherlands over time (up to 30-08-2025).

Fig. 1 provides an overview of patient inclusion. A total of 140 patients underwent EUS with the intent to perform EUS-HGS, of whom 105 were included in the study. Fig. 2A presents an overview of EUS-HGS attempts. Baseline characteristics are described in Table 1 . Most patients were male (55.2%), with a median age of 68 years [IQR: 59–76], classified as WHO performance status I (37.1%) and ASA III (55.2%). Indications for biliary drainage in the palliative setting of malignancy were jaundice ( *n* = 77), cholangitis ( *n* = 26), and optimizing liver function tests to enable palliative chemotherapy ( *n* = 1). In one patient with benign anastomotic stricture after hepaticojejunostomy, cholangitis was the indication to perform biliary drainage. Reasons to perform EUS-HGS instead of conventional biliary drainage methods included gastric-outlet obstruction ( *n* = 40), altered surgical anatomy ( *n* = 21), or failed drainage of the left hepatic duct by ERCP or PTBD ( *n* = 37).

**Table 1:** Baseline characteristics of the total cohort.

	Patients with ≥1 EUS-HGS attempts ( *n* = 105)
Age at diagnosis, median [IQR], years	68 [59–76]
Female sex – *n* (%)	47 (44.8)
ASA classification – *n* (%)	
I	2 (1.9)
II	42 (40.0)
III	58 (55.2)
IV	2 (1.9)
Missing	1 (1.0)
WHO PS – *n* (%)	
0	8 (7.6)
1	39 (37.1)
2	28 (26.7)
3	14 (13.3)
Missing	16 (15.2)
Etiology of biliary obstruction – *n* (%)	
Cholangiocarcinoma	23 (21.9)
Pancreatic cancer	34 (32.4)
Metastatic disease	20 (19.0)
Duodenal cancer	11 (10.5)
Gallbladder cancer	7 (6.7)
Ampullary cancer	2 (1.9)
Gastric cancer	3 (2.9)
Hepatocellular carcinoma	2 (1.9)
Benign	1 (1.0)
Other¥	2 (1.9)
Proximal obstruction (vs distal) – *n* (%)	42 (40.0)
Prior treatment – *n* (%)*	
Resection primary tumor	32 (30.5)
Chemotherapy	38 (36.2)
Radiotherapy	6 (5.7)
Other**	6 (5.7)
Prior GOO treatment – *n* (%)	
Duodenal stent	5 (4.8)
EUS-GE	15 (14.3)
Surgery	7 (6.7)
Ascites present – *n* (%)	30 (28.6)
Altered surgical anatomy – *n* (%)	21 (20.0)
Prior biliary drainage attempts – *n* (%)	
ERCP	56 (53.3)
EUS-GBD	1 (1.0)
Percutaneous	5 (4.8)
Both	11 (10.5)

*EUS-HGS = EUS-guided hepatico-gastrostomy, ASA = American Society of Anaesthesiologists, WHO PS = World Health Organization Performance Status, MBO = malignant biliary obstruction, GOO = gastric outlet obstruction, ERCP = Endoscopic Retrograde Cholangiopancreatography, EUS-GBD = EUS-guided Gallbladder Drainage* .

¥ = Unspecified: Duodenal or ampullary cancer ( *n* = 1), distal cholangiocarcinoma or pancreatic carcinoma ( *n* = 1).

* = Multiple per patient possible.

** = Hepatic arterial infusion chemotherapy ( *n* = 1), irreversible electroporation ( *n* = 1), yttrium embolization ( *n* = 2), histotripsy + dendritic cell therapy ( *n* = 1), endoscopic biliary radiofrequency ablation ( *n* = 1).

### EUS Procedures

Fig. 1 summarizes reasons why EUS-HGS procedures were preliminarily aborted. A total of 107 attempted EUS procedures were performed in 105 patients, with a median procedure time of 62 minutes [IQR: 44.5–87.5]. One patient underwent two EUS procedures without puncture attempts due to insufficient bile duct dilatation, whereas another underwent successful EUS-HGS after an initial EUS showing inadequate ductal dilatation. Overall technical success was achieved in 81/105 patients (77%). The outcomes of the 24 patients without technical success are described in the **Supplementary Text 1** .

Based on the final EUS-HGS attempt per patient, bile ducts could not be safely punctured in 7 of 105 procedures. In the remaining 98 patients, bile duct puncture was successful in 95 (96.9%). Among these patients, deep guidewire insertion was not achieved/performed in three patients. In one patient with ucSEMS in the right bile duct, no stenosis was seen on cholangiogram and the procedure was abandoned. In another patient, interposition of tissue and ascites led the endoscopist to deem the procedure too high risk. In one patient, deep guidewire insertion failed, after which EUS-CDS was successfully performed.

Among the 92 patients in whom deep guidewire insertion was achieved, loss of wire position occurred in two patients, precluding fistula formation and stent insertion. In two additional patients, direct antegrade stenting was deliberately performed instead of EUS-HGS. As a result, fistula formation was performed in 88 patients. In 83 patients (94.3%), fistula formation was performed with a 6 Fr cystotome, whereas in five (5.7%), balloon dilatation was used. Following successful fistula formation, the procedural strategy was intentionally changed in three patients, with antegrade stenting in two cases and a conventional rendezvous approach in one case. These intraprocedural changes reflected adaptations to anatomical or technical considerations. EUS-HGS stent placement was attempted in 85 patients and was successful in 81/85 (95.3%). These and other procedural characteristics are summarized in Table 2 . Technical success did not differ significantly between procedures performed in 2024–2025 and those performed in 2023 or earlier ( *p* = 0.579), as shown in **Fig. S1** .

**Table 2:** Procedural characteristics of the total cohort.

	EUS-HGS attempt ( *n* = 105)
Procedural time, median [IQR], minutes	62 [44.5–87.5]
Biliary stent in-situ at time of EUS-HGS – (%)*	
SEMS	19 (18.1)
SEMS + PTBD	1 (1.0)
Plastic	2 (1.9)**
PTBD	7 (6.7)
EUS-GE in same session – *n* (%)	12 (11.4)
Bile duct puncture attempt – *n* (%)	98/105 (93.3)
Successful bile duct puncture – *n* (%)	95/98 (96.9)
Wire advancement – *n* (%)	92/95 (96.8)
Fistula formation – *n* (%)	88/92 (95.7)
6Fr cystotome	83 (90.2)
Balloon dilatation	5 (9.8)
Stent placement – *n* (%)	86/88 (97.7)
HGS	80 (93.0)
HGS + AGS	1 (1.2)
AGS only	4 (4.7)
RV	1 (1.2)
Stent types among EUS-HGS – *n* (%)	
pcSEMS	50 (61.7)
Additional pigtail	1 (2.0)
Additional fcSEMS	1 (2.0)
ucSEMS + fcSEMS	29 (35.8)
fcSEMS + plastic	1 (1.2)
ucSEMS + plastic	1 (1.2)
Procedural complications – *n* (%)	16 (15.2)

*EUS-HGS = EUS-guided hepatico-gastrostomy, IQR = inter quartile range, SEMS = self-expanding metal stent, PTBD = percutaneous transhepatic biliary drainage, EUS-GE = EUS-guided gastro-enterostomy, fr = French, AGS = antegrade stenting, pcSEMS = partially covered SEMS, ucSEMS = uncovered SEMS, fcSEMS = fully covered SEMS, RV = rendezvous* .

* = Location of the stent or catheter was not collected.

** = Prior EUS-guided gallbladder drainage with LAMS, later switched to double pigtails.

### Stent Characteristics

**Fig. 3:**
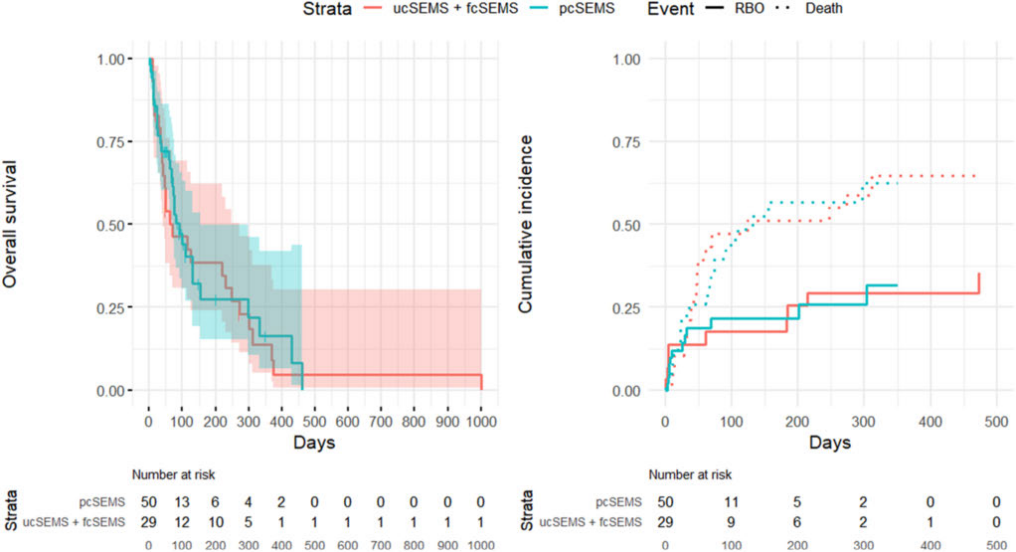
Overall survival curve and cumulative incidence curves of the patients who received pcSEMS or ucSEMS+fcSEMS.

In 50 patients (61.7%), a pcSEMS was placed, with and additional plastic pigtail in one patient and an additional fcSEMS in another. In 29 patients (35.8%), a combination of ucSEMS with fcSEMS was placed. In one patient, a ucSEMS was placed without fcSEMS but with additional plastic pigtail stent. In one patient, an fcSEMS was placed with additional pigtail stent. Stent maldeployment was reported in one patient treated with a pcSEMS, in which the covered segment of the stent was positioned between the stomach and liver, leaving the stent insufficiently extending into the stomach. A repeat procedure was performed two days later during which an fcSEMS was placed across the gap. Meanwhile, the patient remained asymptomatic. Fig. 3 shows the stent types used in the time period.

### Procedural and Postprocedural Complications

During 16 of the 107 procedures, the endoscopist reported a procedural complication. In five procedures, there was clear bile leakage, all treated conservatively with antibiotics and analgesics (GRADE I: 4, GRADE II: 1). In three patients, there was mild procedural bleeding, all treated conservatively (GRADE 0: 1, GRADE I: 2). In three patients, a vessel was punctured, after which the procedure was abandoned in one patient (GRADE IIIA: 1) and successful EUS-HGS after a second puncture in the other two (GRADE 0: 2). In two patients, pneumoperitoneum was identified; one required de-sufflation and intubation but subsequently underwent successful EUS-guided gastro-enterostomy (EUS-GE) and EUS-HGS (GRADE II: 1), while the other was treated conservatively (GRADE I: 1). In one patient, the wire broke in the bile duct but was left in situ (GRADE 0: 1), and in one patient there was mis-deployment of the pcSEMS (GRADE IIIA: 1). In one patient after unsuccessful EUS-HGS, same-session PTBD was performed and postprocedure, there was a clinical suspicion of a pulmonary embolism necessitating CT, but imaging showed no embolism or other complications (GRADE II: 1). In one patient, after fistula formation, the patient became hemodynamically unstable and the procedure was abandoned. Resuscitation was unsuccessful and the patient died due to a cardiac arrest (GRADE V: 1).

The median in-hospital stay duration was 2 days [IQR: 1–7]. Among the patients with bile duct puncture, postprocedure-related complications occurred in 17 of the 98 patients with bile duct puncture (17.3%), encompassing biliary peritonitis in four patients (GRADE II: 2, GRADE IIIA: 2), delayed bleeding in four (GRADE II: 2, GRADE IIIA: 2), cholangitis in three (GRADE II: 2, GRADE IIIA: 1), stent dislocation/migration in three (GRADE IIIA: 3), delirium in one (GRADE II: 1), and bile leakage leading to decompensation cordis and subsequent mortality in one (GRADE V: 1). One patient with a dislocated stent developed biliary peritonitis and subsequently died (GRADE V: 1). There were no procedural complications when EUS-HGS was not attempted.

### Reinterventions and RBO

During a median observed follow-up of 61 days [IQR: 23–131], 23 of the 81 patients (28.4%) developed RBO. **Fig. S2** shows the cumulative incidence curves for RBO development. In two patients, cholangitis occurred after 183 and 1040 days respectively, but no reintervention was indicated as conservative antibiotic treatment was successful. Therefore, in 21 patients, a reintervention was required but was only performed in 18 patients due to inadequate performance status in 3 patients. Thus, clinical success was achieved in 63/81 patients (77.8%). Using a clinical success cut-off of 6 months, where patients who required reintervention after 6 months, clinical success was achieved in 68/81% (84.0%). **Table S2** shows results from univariable and multivariable competing risk regression analysis. No factors were associated with RBO development.

### Survival

Among the 80 patients with malignant disease who underwent successful EUS-HGS, median overall survival was 91 days [IQR: 66–155]. There was no significant difference in median survival between patients who underwent pcSEMS placement and ucSEMS+fcSEMS placement (91 days [IQR: 69–155] vs. 70 days [IQR: 42–273], *p* = 0.81), as shown in Fig. 2B . In univariable analysis, both WHO performance status II-III and ASA classification III-IV were associated with increased risk of death (HR: 3.2, 95%CI: 1.71–6.01 and HR: 1.83, 95%CI: 1.03–3.25, respectively). Multivariable analysis results did not show any significant predictors ( Table 3 ).

**Table 3:** Results from univariable and multivariable cox proportional hazard regression analysis for overall survival after successful EUS-HGS.

	Univariable			Multivariable		
Variable	HR	95%CI	*p* -Value	HR	95%CI	*p* -Value
RBO (vs no RBO)*	1.35	0.77–2.36	0.3	1.07	0.60–1.92	0.809
ucSEMS+fcSEMS (vs pcSEMS)	1.09	0.64–1.86	0.759	0.97	0.54–1.73	0.922
Proximal (vs distal)	1.52	0.89–2.59	0.122	1.48	0.85–2.89	0.169
Female sex (vs Male)	0.75	0.44–1.28	0.292			
Age in years	1.02	1.00–1.04	0.09			
WHO PS II-III (vs 0-I)	3.2	1.71–6.01	**<0.001**			
ASA III-IV (vs I-II)	1.83	1.03–3.25	**0.039**			
Cholangitis	1.35	0.77–2.36	0.296			

*RBO functioning as time-varying covariate.

*HR = Hazard Ratio, CI = Confidence Interval, RBO = recurrent biliary obstruction, SEMS = self-expanding metal stent, ucSEMS = uncovered SEMS, fcSEMS = fully covered SEMS, pcSEMS = partially covered SEMS, WHO PS = World Health Organization Performance Status, ASA = American Society of Anesthesiologists' Physical Status classification system, EUS-HGS = endoscopic ultrasound guided hepatico-gastrostomy* .

### Subgroup Analysis of Same Session EUS-guided Hepatico-gastrostomy and Gastro-enterostomy (GE)

In 12 patients, a same-session EUS-HGS attempt was combined with EUS-GE. Technical success was achieved in nine patients (75%). In seven patients (77.8%), a pcSEMS was placed, in one patient (11.1%) a ucSEMS with fcSEMS, and in one patient (11.1%) a ucSEMS. In three patients (25%), EUS-HGS was not performed: in one patient, the intrahepatic bile duct were not dilated; in another the guidewire could not be advanced distally, prompting conversion to EUS-CDS; and the third patient died from cardiac arrest before completion of the EUS-HGS. Regarding complications, one patient developed pneumoperitoneum during the procedure requiring de-sufflation and intubation. One patient developed RBO requiring repeat ERCP. Technical success of the EUS-GE was 100%. Median survival was 74 days [IQR: 61–NR].

## Discussion

This is the first nationwide, real-world study reporting all consecutive EUS-HGS attempts in clinical practice in the Netherlands, outside of prospective studies. Our data confirm that, in expert centers, EUS-HGS can achieve high technical and clinical success rates with an acceptable safety profile. At the same time, this study underscores that EUS-HGS remains a technically demanding procedure that is associated with substantial rates of technical failure and clinically relevant complications. These findings highlight the need for further standardization of the technique; continued centralization in high-volume expert centers; and more comparative research to optimize patient selection, procedural strategies, and stent choice. In addition, we observed a clear increase in procedural volume over time, accompanied by a shift toward the use of partially covered metal stents, reflecting ongoing technical refinement and growing expertise.

Overall, technically successful EUS-HGS was achieved in 4 out of 5 patients. However, in our cohort, puncture attempts were included as well as intraprocedural changes of strategy toward EUS-AGS or rendezvous-ERCP. This reflects a pragmatic approach that characterizes daily clinical practice more than previous reports. 2 Whenever EUS-HGS stent placement was attempted, technical success rate was 95.3% instead, which does align with the 97.7% technical success rate reported in a recent meta-analysis by Binda et al.. 2 Notably, Binda et al. do not provide a uniform or explicit definition of technical success, which limits direct comparability with real-world cohorts that include aborted or converted procedures. The numbers presented in literature likely overestimate real-world performance due to selective reporting and selection bias, as stated by Binda et al.

The clinical success rate of 78% should be interpreted in the same real-world context. While lower than reported rate of 88.1% [95% CI: 84.7–91.2] in pooled analyses, our outcome definition was different and likely more conservative. 2 In the present study, clinical success was defined as the absence of a need for additional biliary drainage interventions during follow-up, rather than symptom-based or biochemical criteria. Traditional measures for clinical success, such as symptoms or postprocedural bilirubin levels, were difficult to apply retrospectively. As patients frequently underwent EUS-HGS late in their disease process, and to limit their number of visits to the hospital as much as possible, routine checks were seldom indicated. Consequently, symptom relief and bilirubin decrease are less reliable endpoints in retrospective studies, which may partly explain the relatively low clinical success rate observed in our cohort. If we excluded RBO-related reinterventions after 6 months, clinical success improved to 84%.

Both procedural complications and longer-term complications such as RBO are important considerations when evaluating the overall safety and durability of EUS-HGS. In our study, procedural complications encompassing biliary leakage, perforation, pneumoperitoneum, and bleeding occurred in 14.9% of cases. Postprocedural complications, mainly cholangitis or peritonitis, were observed in 17.3%. Despite the complexity of EUS-HGS and the observed postprocedural complication rate, the median hospital stay was 2 days. This may be explained by heterogeneity between participating centers, differences in local discharge practices, and changes in clinical management over the long study period. However, given the retrospective design, we cannot determine with certainty which factors contributed to the relatively short hospital stay. Comparison with existing literature is challenging because complication rates are often reported in aggregate and lack standardized definitions. One case of intraprocedural mortality occurred due to cardiac arrest, but it remains unclear whether this was related to the EUS-HGS or the concomitant EUS-GE. Although a direct comparison with PTBD, the most commonly used alternative to EUS-HGS was not feasible within the scope of this study, it is important to place our findings in the context of the well-documented limitations of PTBD. PTBD is associated with substantial morbidity and mortality, with reported 30-day mortality rates ranging from 17% to 23% and complication rates of 40–70%. 13 Moreover, in many of these patients, PTBD necessitates long-term or even lifelong external biliary drainage. The presence of an external catheter has a profound negative impact on quality of life, as it is associated with persistent discomfort, pain, fatigue, and anxiety, as well as restrictions in daily activities and social functioning. 14 Results from a recent prospective trial on primary percutaneous stenting, without leaving a PTBD catheter in-situ, are promising and warrant further investigation. 15

In the longer term, RBO developed in 28.4% of patients, frequently requiring reintervention or treatment. RBO could develop due to hyperplasia or tumor ingrowth, or due to sludge obstructing the stent. 9 While RBO did not affect overall survival, even after adjusting for stent type and stricture location, it remains clinically relevant because it is associated with repeat procedures and hospital admissions. As quality of life was not formally assessed in this study, its impact can only be inferred indirectly. We were unable to identify any risk factors for RBO, and although some studies have suggested lower recurrence rates with certain stent types, these findings remain inconsistent and are often methodologically limited due to the omission of competing risk regression. 16

Since the first report of Burmester et al. in 2003, 17 the EUS-HGS technique has changed little, with the main innovation being stent design. In Western countries, SEMS are generally preferred over plastic stents. 18 In recent years, the introduction of pcSEMS has led to a widespread implementation across European countries. 4 In the current study, two strategies have been used: pcSEMS has been implemented in recent years, while earlier EUS-HGS used a combination of ucSEMS with additional placement of a fcSEMS. There are currently no reports on the combination of ucSEMS+fcSEMS strategy. We found comparable median overall survival among these groups, and no differences in RBO development. Although pcSEMS are faster and easier to deploy, formal cost-effectiveness analyses are needed to guide optimal stent selection.

In 12 patients, a same-session EUS-GE and EUS-HGS attempt was performed. While EUS-GE was successfully performed in all patients, EUS-HGS was technically successful in nine of the eleven patients who underwent bile duct puncture. Performing both procedures during the same session may be attractive in selected patients, as it can provide single-session palliation of both obstructive problems, avoid multiple procedures or repeated anesthesia, and potentially shorten the time to clinical improvement or oncological treatment. The combination of EUS-GE and EUS-HGS is preferred over surgical alternatives, achieving similar technical and clinical success rates, but fewer complications. 19 For patients with malignant distal biliary obstruction and gastric outlet obstruction, EUS-HGS may be favored over EUS-CDS due to lower complication and RBO rates. 20 However, same-session EUS-GE and EUS-HGS also combines two technically demanding therapeutic EUS procedures, which may increase procedure duration, procedural complexity, and the cumulative risk of adverse events. Therefore, this strategy should be reserved for carefully selected patients and performed by highly experienced therapeutic EUS specialists in centers with appropriate expertise and multidisciplinary support.

This nationwide, multicenter, retrospective study is the first comprehensive evaluation of EUS-HGS implementation in the Netherlands. By systematically including all attempted procedures across expert centers and applying advanced statistical techniques, we aimed to minimalize selection bias and provide a real-world reflection of clinical practice. Competing risk analysis and time-varying covariates are essential for RBO and overall survival regression analysis. We deliberately excluded cases included in the EPSILON trial and ongoing studies to avoid the risk of double publication, while we acknowledge the risk of selection bias. 9 Some limitations should be acknowledged. Firstly, long-term follow-up was incomplete for several patients referred back to their primary hospitals. Secondly, our definition of success, focused on objective, procedure-related outcomes, may not fully capture patient-reported outcomes or quality of life, which are increasingly relevant for clinical decision-making. Thirdly, a learning curve analysis could not be performed, as the number of procedures per center was insufficient to allow reliable assessment. Lastly, the indication for EUS-HGS has evolved over time, from a last-resort option to a well-established procedure within the therapeutic repertoire of expert centers. The absolute number of EUS-HGS procedures performed per center remained modest, which may influence both the performance and complication rates. Consequently, the observed rates of technical failure and complications in our cohort may, at least in part, reflect this low procedural density rather than intrinsic limitations of the technique itself. This finding reinforces the importance of structured training and ongoing centralization.

In conclusion, EUS-HGS is a feasible rescue biliary drainage strategy with acceptable safety when performed in expert centers. In this nationwide real-world cohort, technical and clinical success were slightly lower than previously reported, likely reflecting routine clinical practice rather than highly selected series. Stent type did not influence outcomes. Further prospective studies are needed to optimize patient selection and procedural strategy.

## Supplementary Material

Ergänzendes Material Supplementary Material
