# Supplementary material for: Bridging the Gap between Literature and Practice: Nationwide Outcomes of Endoscopic Ultrasound-guided Hepatico-gastrostomy
Source: Endosc Int Open. 2026 Jun 25;14:a28849422. doi: 10.1055/a-2884-9422 (PMC13305367; doi:10.1055/a-2884-9422)

Supplementary Files for

"Bridging the Gap between Literature and Practice: nationwide outcomes of endoscopic ultrasound-guided hepatico-gastrostomy"

by

David M. de Jong, Lydi M.J.W. van Driel, Jurriën G.P. Reijnders, Akin Inderson, Jan Werner Poley, Roy L.J. van Wanrooij, Rogier P. Voermans, Paul Didden, Thomas R. de Wijkerslooth, Foke van Delft, Niels G. Venneman, Robert C. Verdonk, Johanna P. van Nes, Marco J. Bruno, Willem J. Lammers

Endosc Int Open | DOI 10.1055/a-2884-9422 | © The Author(s).

Supplementary Text 1

In 24 patients, no technically successful EUS-HGS was performed. The following interventions were performed in these patients:

- Same session EUS-guided antegrade stenting: 4
- Same session EUS-guided rendez-vous: 1
- Same session switch to EUS-guided choledochoduodenostomy: 1
- Same session switch to ERCP: 3
- Percutaneous transhepatic biliary drainage by catheter placement: 6
- Percutaneous self-expanding metal stent placement: 1
- No further interventions performed: 7
- Procedural mortality precluding further biliary drainage procedures: 1

**Center**	**Up to which date included**
Erasmus MC University Medical Center	26-08-2025
Amsterdam University Medical Center	23-07-2025*
Leiden University Medical Center	01-07-2025
Maastricht University Medical Center+	13-06-2025
University Medical Center Utrecht	23-07-2025
Radboud University Medical Center	20-06-2025
Medisch Spectrum Twente	06-05-2025
St. Antonius Hospital Nieuwegein	10-08-2025
Antoni van Leeuwenhoek Hospital	28-08-2025*

Table S1. End of data collection period per center

*EPSILON patients and prospective study are excluded from analysis, but included in the Figure

	**Univariable**			**Multivariable**		
**Variable**	**sHR**	**95%CI**	**p-value**	**sHR**	**95%CI**	**p-value**
ucSEMS+fcSEMS (vs pcSEMS)	1.17	0.51 – 2.71	0.71	1.03	0.42 – 2.48	0.953
Proximal (vs distal)	1.91	0.86 – 4.28	0.114	1.95	0.81 – 4.69	0.137
Female sex (vs Male)	1.26	0.57 – 2.79	0.567			
Age in years	1.00	0.97 – 1.02	0.881			
WHO PS II-III (vs 0-I)	0.47	0.16 – 1.35	0.161			
ASA III-IV (vs I-II)	0.69	0.31 – 1.54	0.367			
Cholangitis	0.85	0.34 – 2.14	0.733			

Table S2. Results from univariable and multivariable competing risk regression analysis for RBO occurrence after successful EUS-HGS

HR = Hazard Ratio, CI = Confidence Interval, RBO = Recurrent Biliary Obstruction, SEMS = self-expanding metal stent, ucSEMS = uncovered SEMS, fcSEMS = fully covered SEMS, pcSEMS =

partially covered SEMS, WHO PS = World Health Organization Performance Status, ASA = American Society of Anesthesiologists' Physical Status classification system, EUS-HGS = endoscopic ultrasound guided hepaticogastrostomy

Supplementary Figure 1

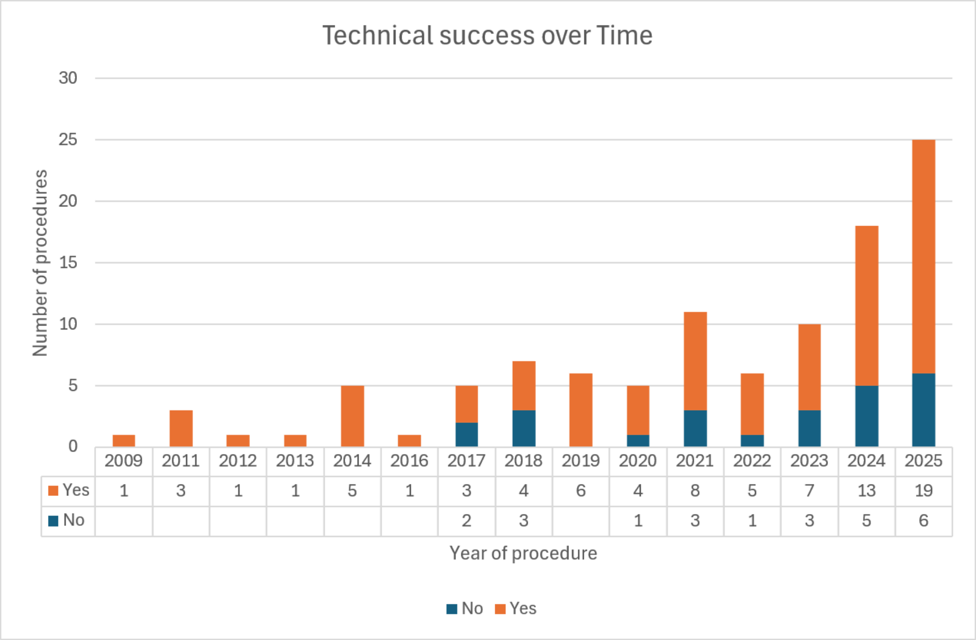

Supplementary Figure 2

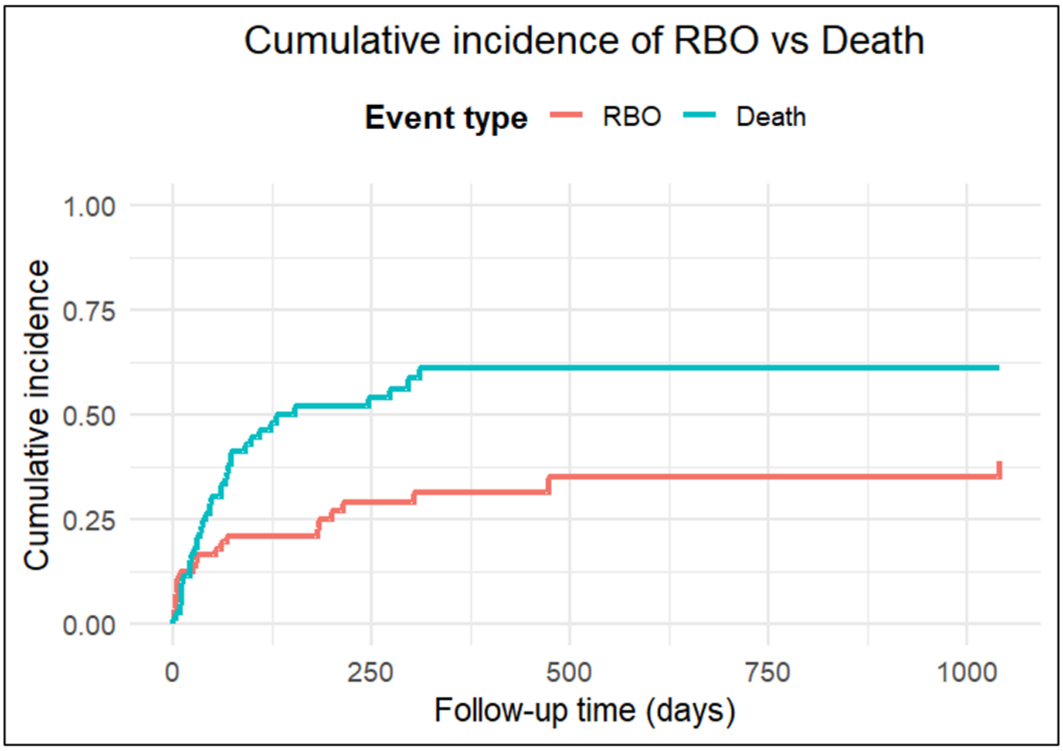

Supplement: Supplementary file 2 — Ergänzendes Material [file 10-1055-a-2884-9422_28863015.pdf]
